# Supplementary material for: IKAROS levels are associated with antigen escape in CD19- and CD22-targeted therapies for B-cell malignancies
Source: Nat Commun. 2025 Apr 23;16:3800. doi: 10.1038/s41467-025-58868-2 (PMC12019336; doi:10.1038/s41467-025-58868-2)
Supplement: Supplementary file 1 — Supplementary Information [file 41467_2025_58868_MOESM1_ESM.pdf]

Supplementary Information: IKAROS levels are associated with antigen escape in CD19- and CD22-targeted therapies for B-cell malignancies

Pablo Domizi[*1], Jolanda Sarno[1,2,3], Astraea Jager[1], Milton Merchant[1], Kaithlen Zen B Pacheco[4], Sean A. Yamada-Hunter[4], Maria Caterina Rotiroti[5], Yuxuan Liu[1], Reema Baskar[6], Warren D. Reynolds[4], Brian J. Sworder[7], Bita Sahaf[4], Sean C. Bendall[6], Charles G. Mullighan[8], Ash A. Alizadeh[7,9,10], Allison B. Leahy[11,12], Regina M. Myers[11,12], Bonnie Yates[13], Hao-Wei Wang[14], Nirali N. Shah[13], Robbie G. Majzner[5], Crystal L. Mackall[4], Stephan A. Grupp[11,12], David M. Barrett[15], Elena Sotillo[4] and Kara L. Davis[*1,4]

[1] Department of Pediatrics, Hematology, Oncology, Stem Cell Transplant and Regenerative Medicine, Stanford University, Stanford, CA, USA.
[2] Tettamanti Center, Fondazione IRCCS San Gerardo dei Tintori, Monza, Italy.
[3] School of Medicine and Surgery, University of Milano-Bicocca, 20126 Milan, Italy.
[4] Center for Cancer Cell Therapy, Stanford Cancer Institute, Stanford University School of Medicine, Stanford, CA, USA.
[5] Department of Pediatric Oncology, Dana-Farber Cancer Institute, Boston, MA, USA.
[6] Department of Pathology, Stanford University, Stanford, CA, USA.
[7] Division of Oncology, Department of Medicine, Stanford University School of Medicine, Stanford, CA, USA.
[8] Department of Pathology, St. Jude Children's Research Hospital, Memphis, TN, USA.
[9] Institute for Stem Cell Biology and Regenerative Medicine, Stanford University, Stanford, CA, USA.
[10] Stanford Cancer Institute, Stanford University, Stanford, CA, USA.
[11] Division of Oncology, Children's Hospital of Philadelphia, Philadelphia, PA, USA.
[12] Department of Pediatrics, Perelman School of Medicine, University of Pennsylvania, Philadelphia, PA, USA.
[13] Pediatric Oncology Branch, Center for Cancer Research, National Cancer Institute, National Institutes of Health, Bethesda, MD, USA.
[14] Laboratory of Pathology, National Cancer Institute, National Institutes of Health, Bethesda, MD, USA.
[15] Kite Pharma, Santa Monica, CA, USA.

*Correspondence: kardavis@stanford.edu; domizi@stanford.edu

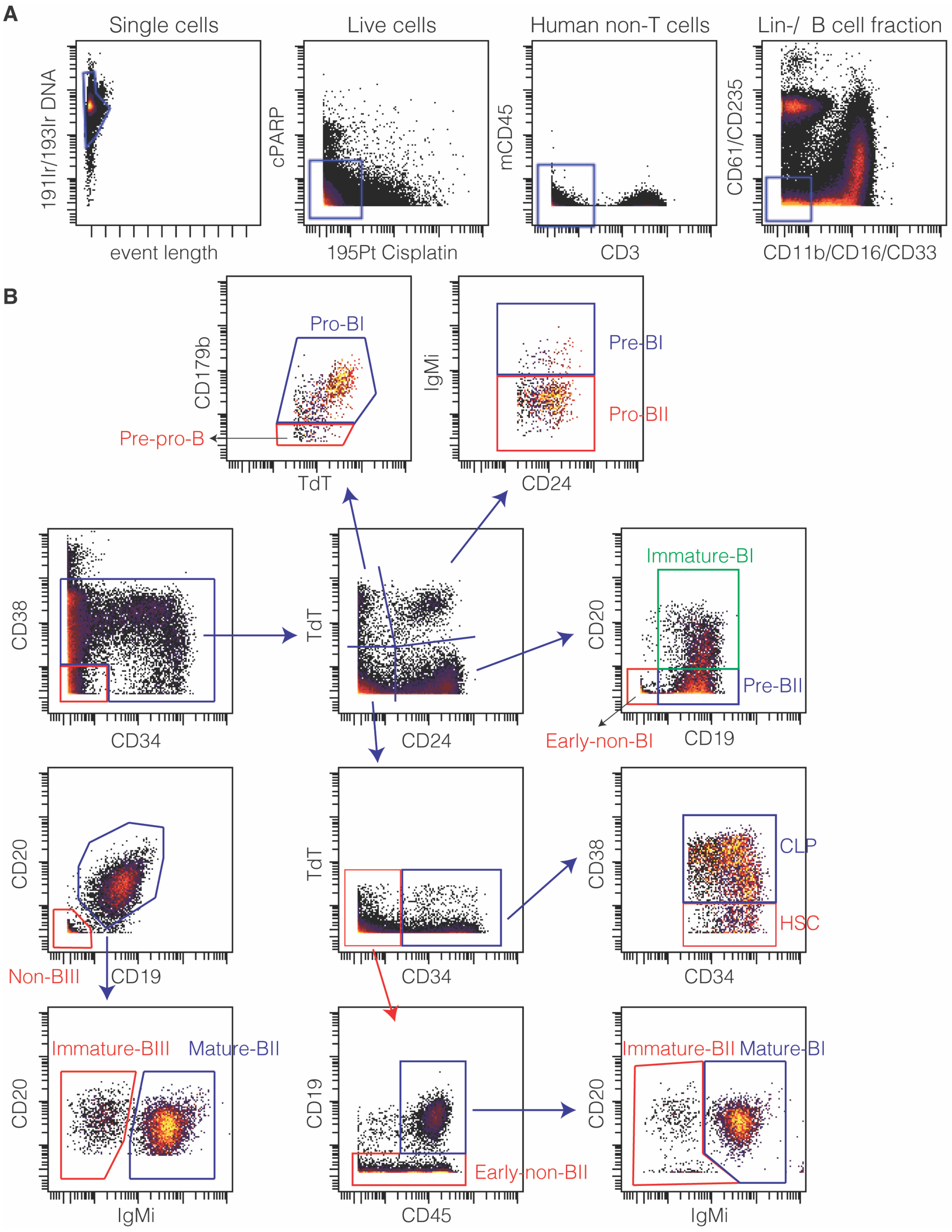
A
Single cells
191Ir/193Ir DNA
event length
Live cells
cPARP
195Pt Cisplatin
Human non-T cells
mCD45
CD3
Lin-/ B cell fraction
CD61/CD235
CD11b/CD16/CD33
B
Pro-BI
CD179b
Pre-pro-B
TdT
Pre-BI
IgMi
Pro-BII
CD24
CD38
CD34
TdT
CD24
Immature-BI
CD20
Pre-BII
Early-non-BI
CD19
CD20
Non-BIII
CD19
TdT
CD34
CLP
CD38
HSC
CD34
Immature-BIII
Mature-BII
CD20
IgMi
CD19
Early-non-BII
CD45
Immature-BII
Mature-BI
CD20
IgMi

Supplementary Figure 1: Manual gating strategy.
(**A**) Gating strategy for lineage-negative/ B cell (Lin-/ B+) fraction enrichment from healthy BM or leukemia cells. This is the starting population for all subsequent analyses of normal and leukemic samples. (**B**) Gating strategy to identify 12 subpopulations of B lymphopoiesis and 3 non-B populations among Lin-/ B+ fraction from healthy BM.

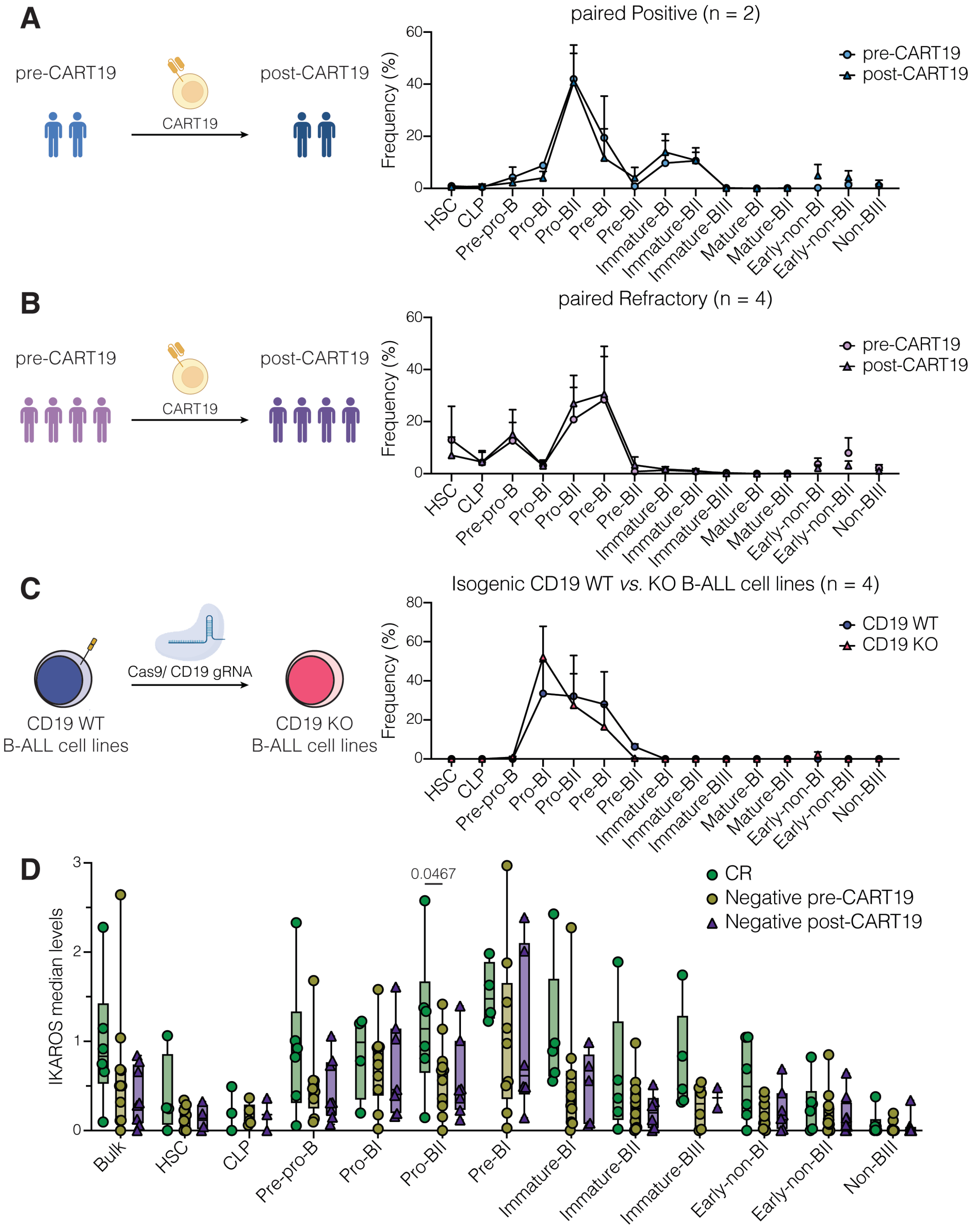

A
pre-CART19
CART19
post-CART19
paired Positive (n = 2)
Frequency (%)
pre-CART19
post-CART19
HSC
CLP
Pre-pro-B
Pro-BI
Pro-BII
Pre-BI
Pre-BII
Immature-BI
Immature-BII
Immature-BIII
Mature-BI
Mature-BII
Early-non-BI
Early-non-BII
Non-BIII
B
paired Refractory (n = 4)
C
Cas9/ CD19 gRNA
CD19 WT
B-ALL cell lines
CD19 KO
B-ALL cell lines
Isogenic CD19 WT vs. KO B-ALL cell lines (n = 4)
CD19 WT
CD19 KO
D
IKAROS median levels
0.0467
CR
Negative pre-CART19
Negative post-CART19
Bulk

Supplementary Figure 2: Enrichment in early-non-BI population is restricted to CD19^{neg} relapse patients.

(A - C) Developmental classification of pre-CART19 and paired post-CD19^{pos} relapse (A; n = 2), refractory (B; n = 4) samples, and isogenic CD19 WT or KO B-ALL cell lines (C; n = 4). Schematic representation of the patient cohort and CD19KO cell lines created in BioRender. Domizi, P. (2025) https://BioRender.com/8ud04e8.

(D) IKAROS median levels in bulk and different B-cell developmental populations from pre-CART19 patients that achieved durable CR (n = 6), suffered CD19^{neg} relapse (n = 11), and post-CD19 loss (n = 8), respectively.

Curves in (A - C) show mean ± SEM. Boxes in (M) extend from the 25th to the 75th percentiles, with a line in the middle representing the median and whiskers extending from the minimum to the maximum values. Statistical tests were paired two-way ANOVA followed by Šidák's multiple comparisons tests (A - C); and two-way ANOVA followed by Tukey's multiple comparisons tests (D).

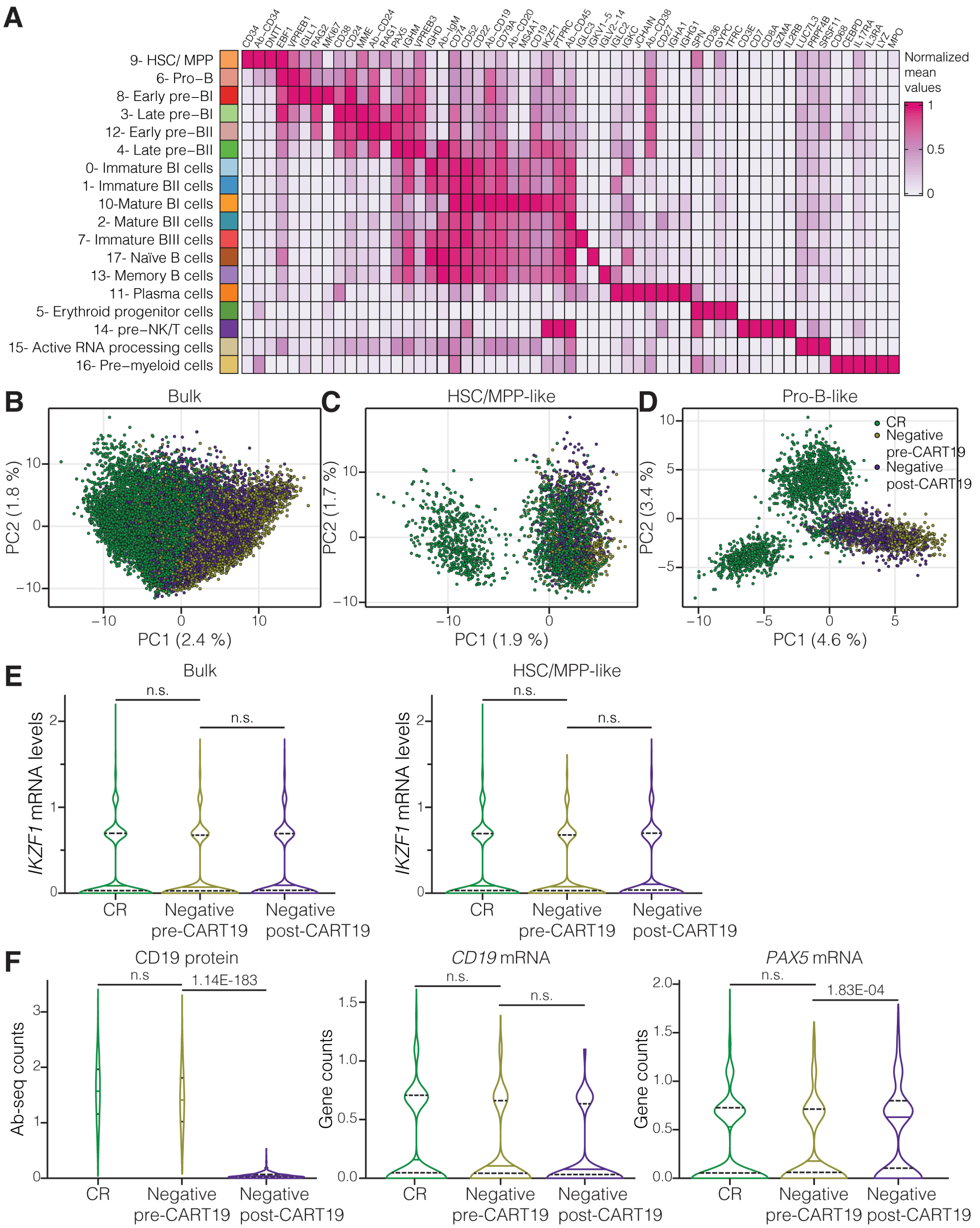

A
9- HSC/ MPP
6- Pro−B
8- Early pre−BI
3- Late pre−BI
12- Early pre−BII
4- Late pre−BII
0- Immature BI cells
1- Immature BII cells
10-Mature BI cells
2- Mature BII cells
7- Immature BIII cells
17- Naïve B cells
13- Memory B cells
11- Plasma cells
5- Erythroid progenitor cells
14- pre−NK/T cells
15- Active RNA processing cells
16- Pre−myeloid cells
Normalized mean values
B
Bulk
PC1 (2.4 %)
PC2 (1.8 %)
C
HSC/MPP-like
PC1 (1.9 %)
PC2 (1.7 %)
D
Pro-B-like
PC1 (4.6 %)
PC2 (3.4 %)
CR
Negative pre-CART19
Negative post-CART19
E
Bulk
HSC/MPP-like
IKZF1 mRNA levels
n.s.
CR
Negative pre-CART19
Negative post-CART19
F
CD19 protein
Ab-seq counts
n.s
1.14E-183
CD19 mRNA
Gene counts
n.s.
PAX5 mRNA
1.83E-04

Supplementary Figure 3: Pre-treatment low *IKZF1* expression and unique gene expression signature in pro-B-like B-ALL cells from CD19^{neg} relapsed patients.

(**A**) Mean expression of selected significant markers (FDR < 0.05) for each healthy cluster. Differential expression analysis (Wilcoxon Rank Sum test) was performed between cells from one cluster against cells from all the other clusters. Mean values of each marker were scaled from 0 to 1, to facilitate the visualization of changes in marker expression across different populations.

(**B - D).** Principal component analysis based on genes differentially expressed between bulk (B), HSC/ MPPC-like (C), and pro-B-like (D) B-ALL cells from pre-CART19 patients that will achieve durable CR or suffer CD19^{neg} relapse.

(E) *IKZF1* gene expression in bulk (left) and HSC/MPP-like (right) B-ALL cells.

(**F**) CD19 protein (left), *CD19* (middle), and *PAX5* (right) gene expression in pro-B-like B-ALL cells.

Violin plots in (E - F) show median (solid line) and 25th and 75th quantile (dash lines). Statistical test used was Wilcoxon rank sum test followed by Bonferroni's multiple comparisons test (E). Not significant (n.s.), P>0.05.

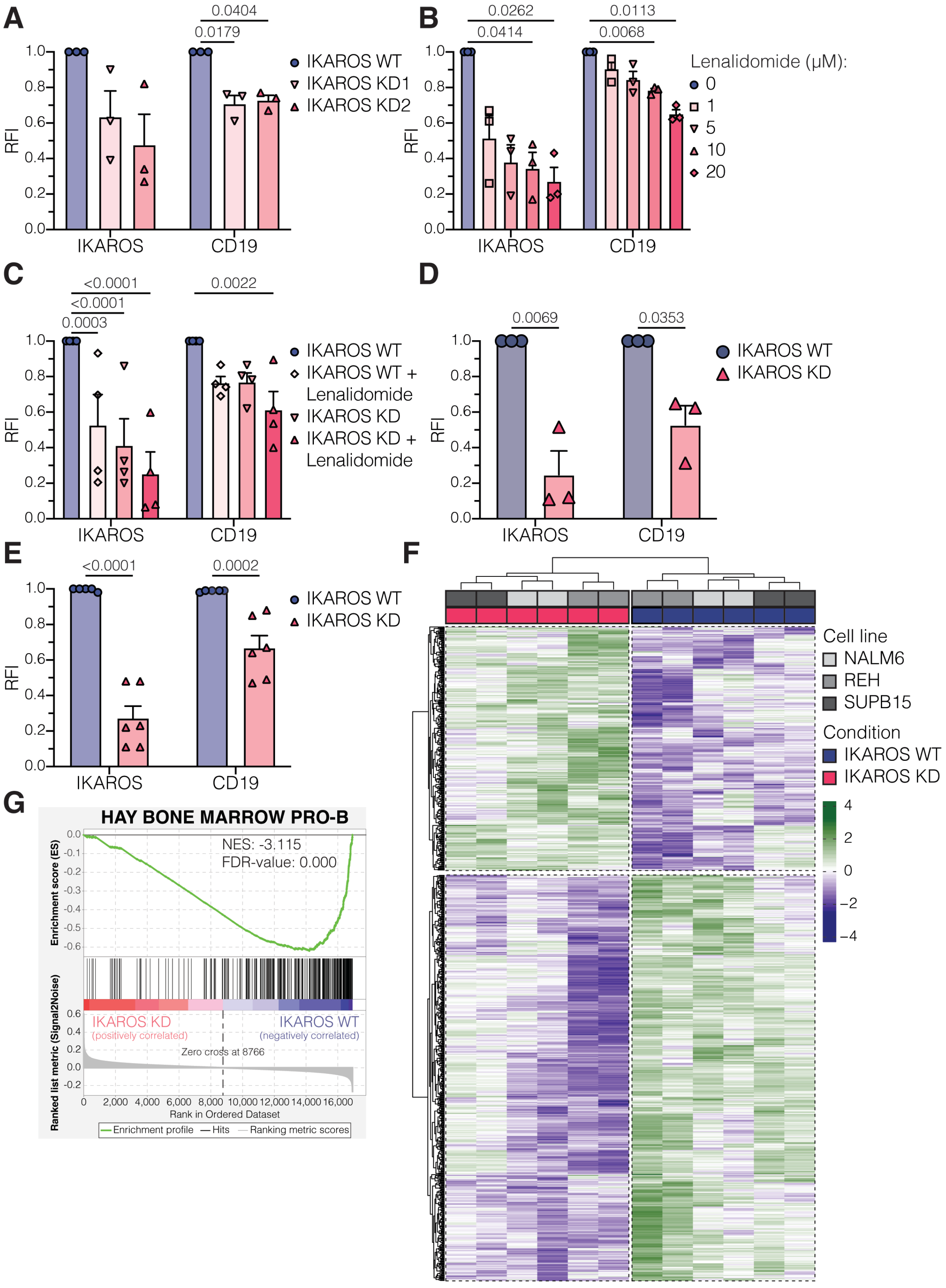

A
0.0404
0.0179
RFI
IKAROS
CD19
IKAROS WT
IKAROS KD1
IKAROS KD2
B
0.0262
0.0414
0.0113
0.0068
Lenalidomide (μM):
0
1
5
10
20
C
<0.0001
<0.0001
0.0003
0.0022
IKAROS WT
IKAROS WT + Lenalidomide
IKAROS KD
IKAROS KD + Lenalidomide
D
0.0069
0.0353
IKAROS WT
IKAROS KD
E
<0.0001
0.0002
IKAROS WT
IKAROS KD
F
Cell line
NALM6
REH
SUPB15
Condition
IKAROS WT
IKAROS KD
G
HAY BONE MARROW PRO-B
NES: -3.115
FDR-value: 0.000
Enrichment score (ES)
Ranked list metric (Signal2Noise)
IKAROS KD
(positively correlated)
IKAROS WT
(negatively correlated)
Zero cross at 8766
Rank in Ordered Dataset
Enrichment profile
Hits
Ranking metric scores

Supplementary Figure 4: IKAROS modulates CD19 surface expression in other B-cell malignancies.

(**A - C**) IKAROS and CD19 median expression values in LBCL cell lines (OCI-Ly1, OCI-Ly7, SUDHL6) transduced with lentivirus expressing scrambled or short hairpin RNA (shRNA) against *IKZF1* (A), treated with increasing doses of lenalidomide (B), or combining shRNA with or without lenalidomide treatment (C). Proteins were measured by flow cytometry and normalized to scrambled transduced (A), DMSO-treated (B), or scrambled transduced and DMSO-treated (C) cells. RFI = relative fluorescence intensity.

(**D**) IKAROS and CD19 median expression in isogenic IKAROS WT or KD CLL cell lines (CI, JVM-2, WA-OSEL). Proteins were measured by flow cytometry and normalized to WT condition. RFI = relative fluorescence intensity.

(**E**) IKAROS and CD19 median expression in isogenic IKAROS WT or KD B-ALL cell lines (NALM6, REH, SUP-B15) used for ATAC-seq and RNA-seq experiments. Proteins were measured by flow cytometry and normalized to WT condition. RFI = relative fluorescence intensity.

(F) Z-score of differentially expressed genes between isogenic IKAROS WT and KD B-ALL cells.

(**G**) GSEA for Hay Bone Marrow Pro-B gene signature[75] in IKAROS WT and KD B-ALL cells.

Bar plots in (A - E) show mean ± SEM. Statistical tests used were two-way ANOVA followed by Tukey's multiple comparisons test (A - C); and one-way ANOVA followed by Šidák's multiple comparisons test (D - E). *P<0.05, **P<0.01, ***P<0.001, ****P< 0.0001.

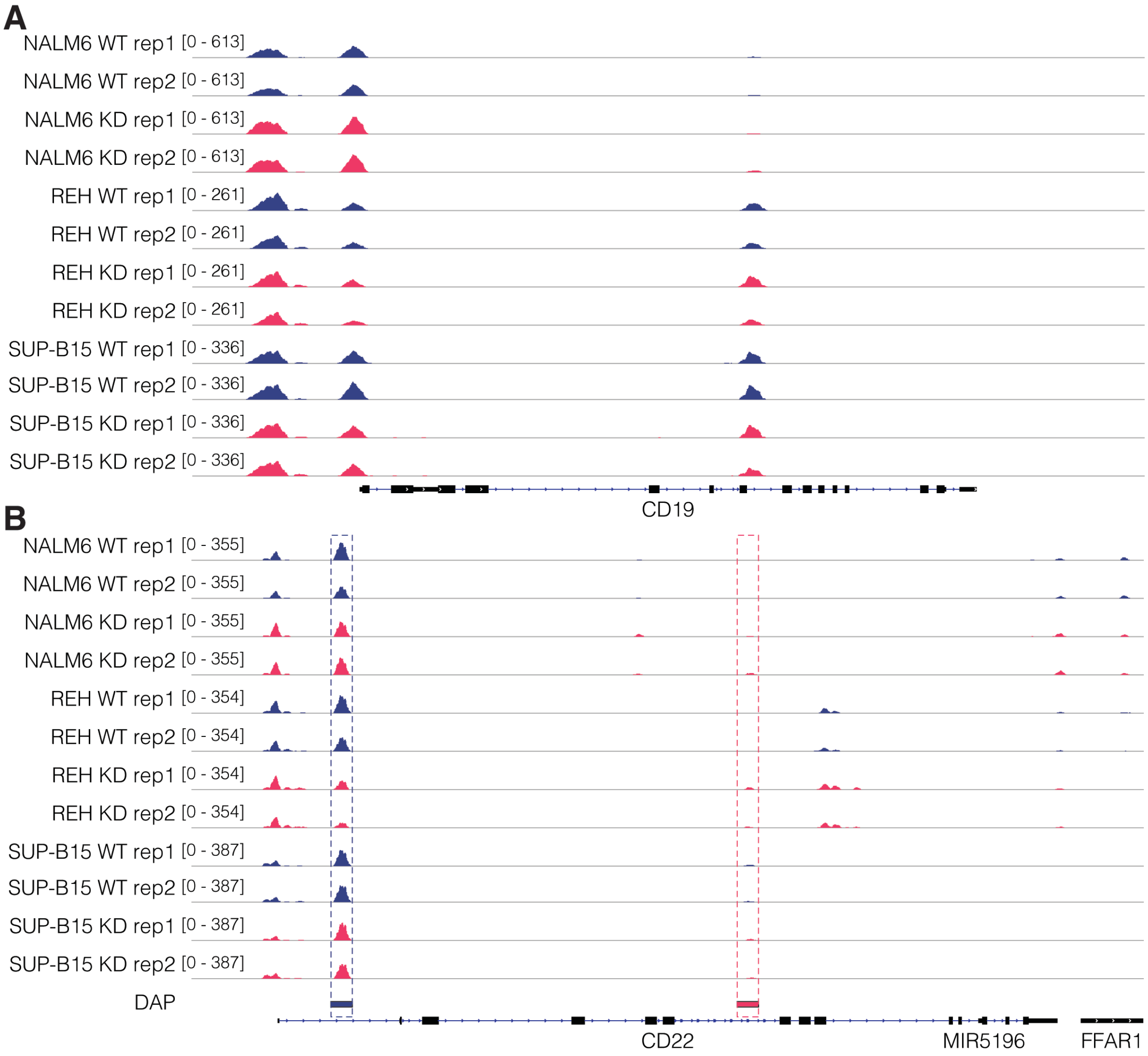

Supplementary Figure 5: Low levels of IKAROS promote changes in chromatin accessibility at the CD22 gene, while not affecting the CD19 gene.
(A - B) ATAC-seq chromatin accessibility track to *CD19* (A) and *CD22* (B) promoter and gene from isogenic IKAROS WT and KD B-ALL cell lines. Experiment was performed in 3 B-ALL cell lines (NALM6, REH, SUP-B15) in duplicate. DAP stands for differentially accessible peaks in IKAROS WT (blue) or KD (red) conditions.

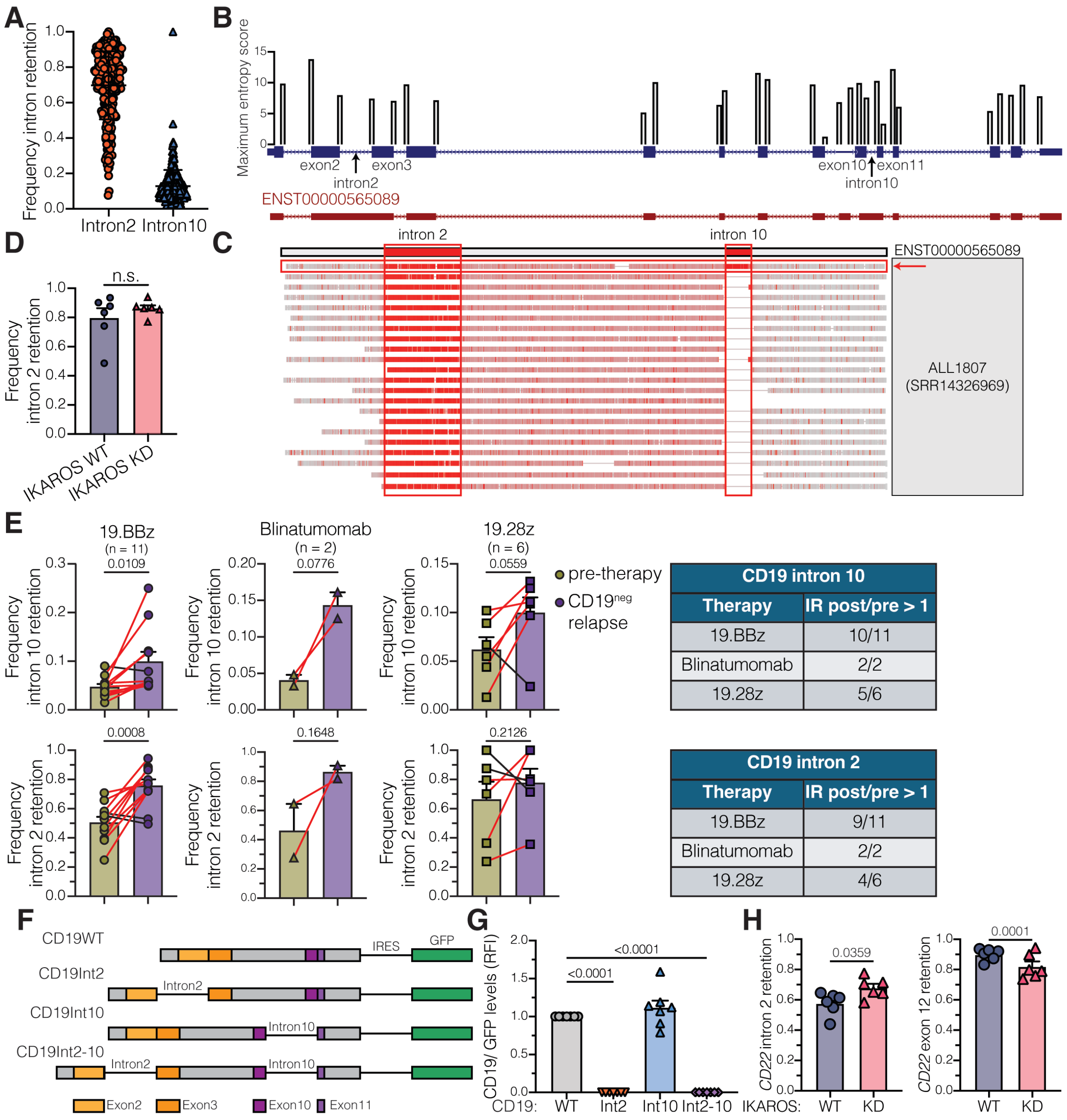

CD19 intron 10	
**Therapy**	**IR post/pre > 1**
19.BBz	10/11
Blinatumomab	2/2
19.28z	5/6

CD19 intron 2	
**Therapy**	**IR post/pre > 1**
19.BBz	9/11
Blinatumomab	2/2
19.28z	4/6

Supplementary Figure 6: *CD19* intron 10 is co-retained with intron 2.
(**A**) Frequency of *CD19* intron 2 and intron 10 retention in *de novo* and relapsed B-ALL patient samples (n = 282) from TARGET ALL project.
(**B**) Schematic representation of human *CD19* gene and ENST00000565089 transcript, which retained both intron 2 and intron 10. Bars on top of *CD19* gene correspond to splice site strengths (maximum entropy score), calculated using MaxEntScan. Splice sites surrounding intron 10 are of similar strength to the rest of introns, suggesting that intron 10 retention does not happen by unintended mistakes by the splicing machinery.
(**C**) Direct RNA long-reads from one B-ALL PDX[76] (Accession number: SRR14326969) were aligned to *ENST00000565089* using the online version of the BLAST algorithm. Reads containing intron 2 or intron 10 were selected for illustration. Interestingly, the only long-read containing intron 10, also retains intron 2.
(**D**) Frequency of intron 2 retention in *CD19* mRNA in IKAROS WT or KD B-ALL cells (n=6).
(**E**) Frequency of intron 10 and 2 retention in *CD19* mRNA in pediatric B-ALL patients treated with 19.BBz CAR T cells[11,14] (n=11), adult B-ALL patients treated with blinatumomab[13] (n=2), and adult LBCL patients treated with 19.28z CAR T cells[31,32] (n=6). Paired samples that show higher retention of introns 2 or 10 following CD19^{neg} relapse are indicated with red lines. The tables summarize the number of patients exhibiting higher retention of introns 2 or 10 after CD19^{neg} relapse compared to the total number of patients per CD19-targeted therapy.
(**F**) Schematic illustration of *CD19* gene constructs without intron 2 or intron 10 (WT), with intron 2 (Int2), intron 10 (Int10), or both intron 2 and intron 10 (Int2-10) retention. CD19 KO B-ALL cell lines (697, NALM6, NALM16, REH) were transduced with retrovirus expressing different CD19 isoforms. Bicistronic RNA was used to co-deliver CD19 isoforms and GFP expression.
(G) Surface CD19 and GFP median levels. Proteins were measured by flow cytometry (n = 7), CD19 levels were normalized over GFP levels for each condition, and then further normalized by CD19/ GFP ratio from CD19 WT isoform. RFI = relative fluorescence intensity.
(**H**) Frequency of intron 2 (left) or exon 12 (right) retention in *CD22* mRNA in IKAROS WT or KD B-ALL cells.
Error bars in (A) show mean ± SEM. Bar plots in (D - E), and (G - H) show mean ± SEM.
Statistical tests used were multivariate analysis of transcript splicing (H), one-tailed paired t-test (D - E), and one-way ANOVA followed by Dunnett's multiple comparisons tests (G). Not significant (n.s.), P>0.05.

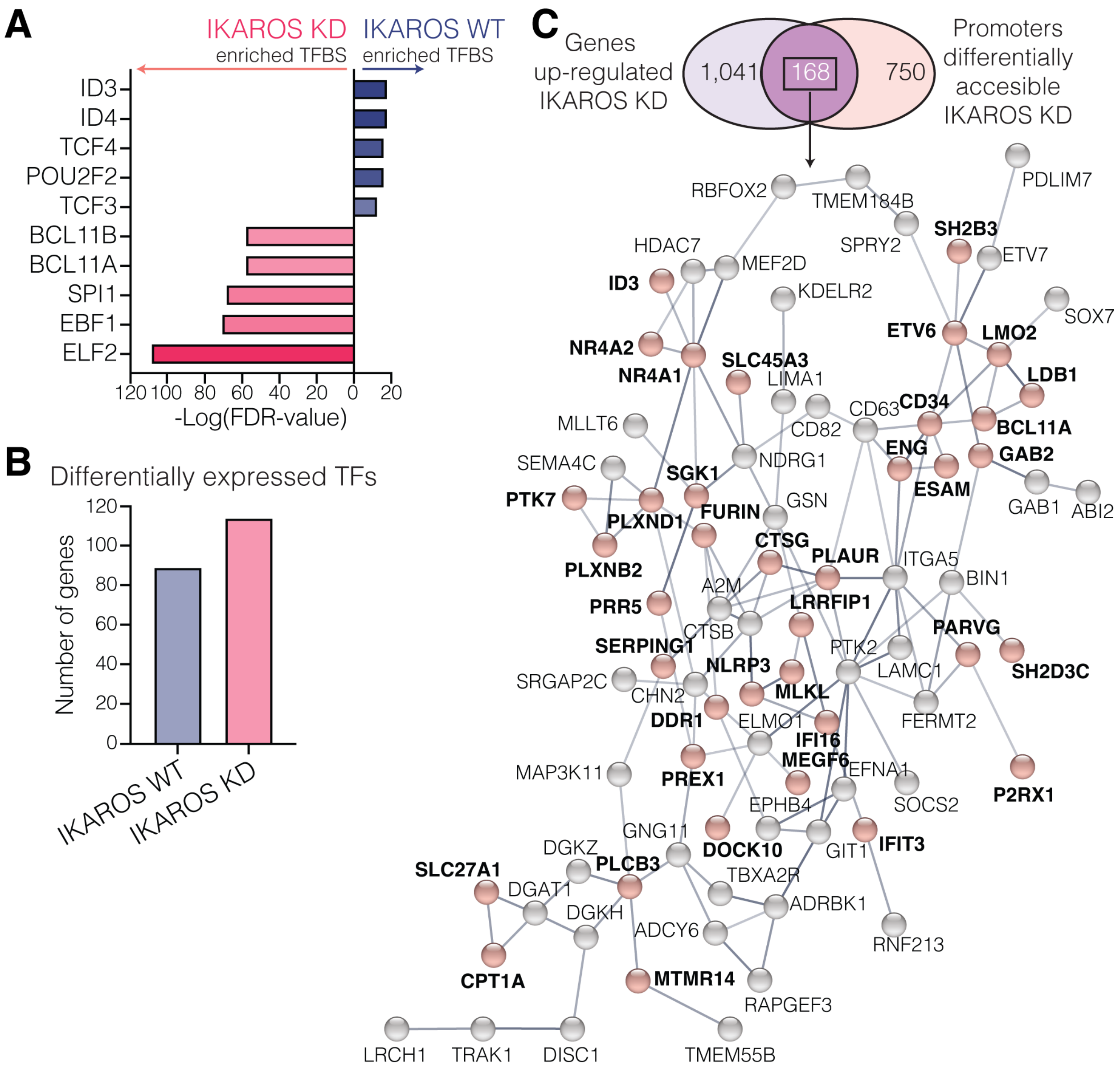

Supplementary Figure 7: Low levels of IKAROS induces expression of a non-B lineage gene network.

(**A**) Top 10 transcription factor binding sites (TFBS) enriched in differentially open peaks.

(**B**) Number of differentially up-regulated transcription factor encoding genes in IKAROS WT or KD B-ALL cells.

(**C**) Venn diagram between genes up-regulated and genes whose promoters have differentially accessible peaks in IKAROS KD B-ALL cells. STRING (Search Tool for Retrieval of Interacting Genes/Proteins) protein-protein interaction network of genes up-regulated and with more accessible promoters in IKAROS KD cells. Node represents gene and line thickness is proportional to the confidence of interaction. Red nodes correspond to genes associated with non-B cell lineage.

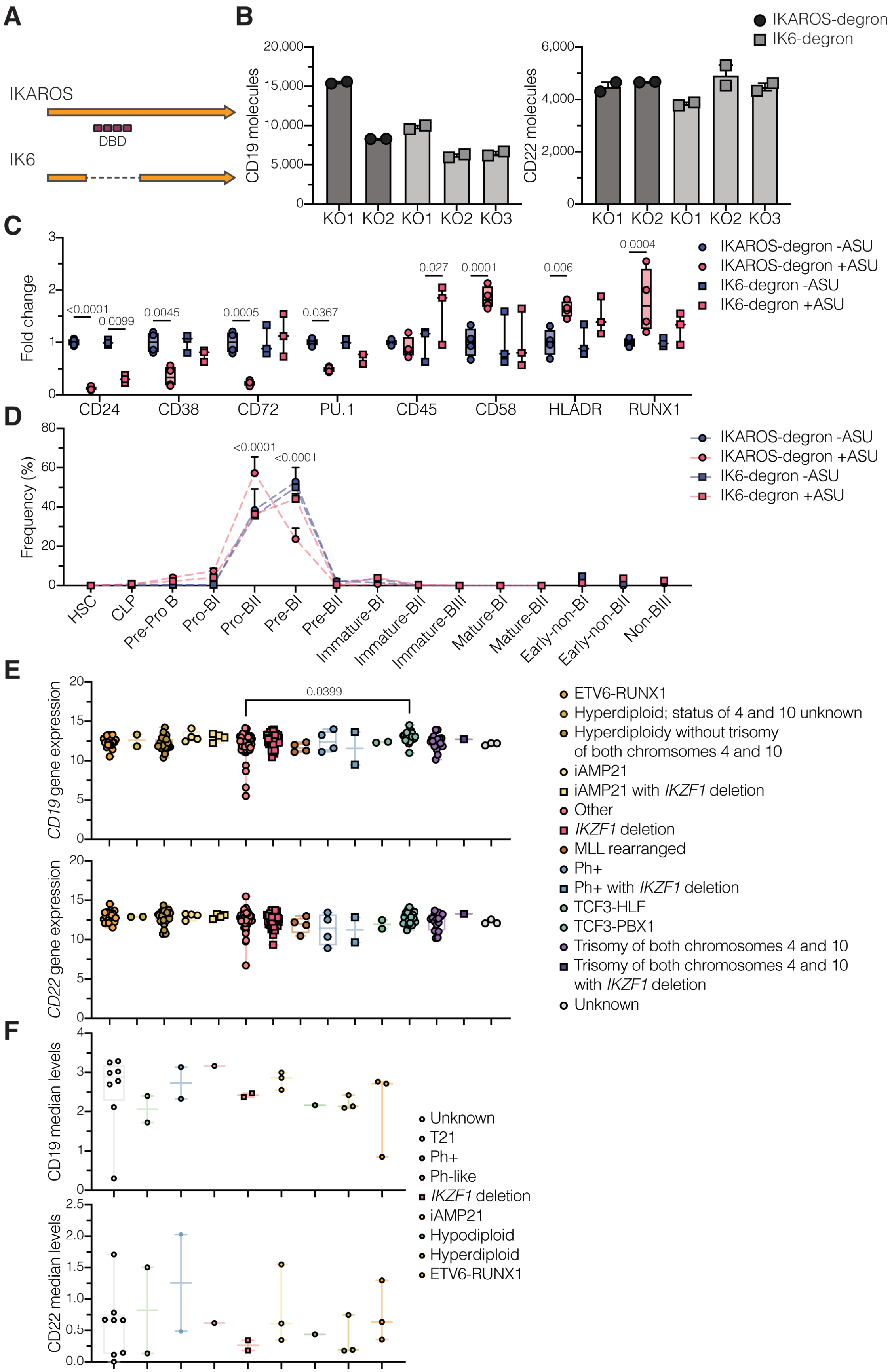

A
IKAROS
DBD
IK6
B
IKAROS-degron
IK6-degron
CD19 molecules
CD22 molecules
KO1 KO2 KO1 KO2 KO3
C
Fold change
CD24 CD38 CD72 PU.1 CD45 CD58 HLADR RUNX1
IKAROS-degron -ASU
IKAROS-degron +ASU
IK6-degron -ASU
IK6-degron +ASU
D
Frequency (%)
HSC CLP Pre-Pro B Pro-BI Pro-BII Pre-BI Pre-BII Immature-BI Immature-BII Immature-BIII Mature-BI Mature-BII Early-non-BI Early-non-BII Non-BIII
E
CD19 gene expression
CD22 gene expression
ETV6-RUNX1
Hyperdiploid; status of 4 and 10 unknown
Hyperdiploidy without trisomy of both chromosomes 4 and 10
iAMP21
iAMP21 with IKZF1 deletion
Other
IKZF1 deletion
MLL rearranged
Ph+
Ph+ with IKZF1 deletion
TCF3-HLF
TCF3-PBX1
Trisomy of both chromosomes 4 and 10
Trisomy of both chromosomes 4 and 10 with IKZF1 deletion
Unknown
F
CD19 median levels
CD22 median levels
Unknown
T21
Ph+
Ph-like
IKZF1 deletion
iAMP21
Hypodiploid
Hyperdiploid
ETV6-RUNX1

Supplementary Figure 8: Deletion of IKAROS DNA binding domains abrogate modulation of CD19 and CD22 surface expression.

(**A**) Schematic representation of IKAROS deletion used to generate the IK6-degron model. DBD stands for DNA binding domains.

(B) Baseline CD19 (left) and CD22 (right) molecule numbers on the cell surface of IKAROS-degron (KO1 and KO2; circles) and IK6-degron (KO1, KO2, and KO3; squares) models. Experiment was performed in duplicate.

(C) IKAROS- and IK6-degron models were treated with DMSO (-ASU; n = 4), or 10 µM asunaprevir (+ASU; n = 4) for 7 days. Protein profile was measured by CyTOF and normalized to -ASU condition.

(D) Developmental classification of samples in (C). Frequencies of Pro-BII and Pre-BI populations were statistically different (p-value < 0.0001) in the IKAROS-degron +ASU condition compared to the other conditions.

(E) *CD19* (top) and *CD22* (bottom) vst counts across different B-ALL genomic subtypes with or without *IKZF1* deletions in B-ALL patient samples (n = 282) from TARGET ALL project.

(F) Bulk CD19 (top) and CD22 (bottom) median levels in pre-CART19 samples (Figure 1A) based on their cytogenetic classification.

Bar plots in (B) show mean ± SEM. Boxes in (C) and (E – F) extend from the 25th to the 75th percentiles, with a line in the middle representing the median and whiskers extending from the minimum to the maximum values. Curves in (D) show mean ± SEM. Statistical tests used were Kruskal-Wallis followed by Dunn’s multiple comparisons tests (B) and (F); two-way ANOVA followed by Tukey’s multiple comparisons tests (C - D); and one-way ANOVA followed by Tukey’s multiple comparisons tests (E).

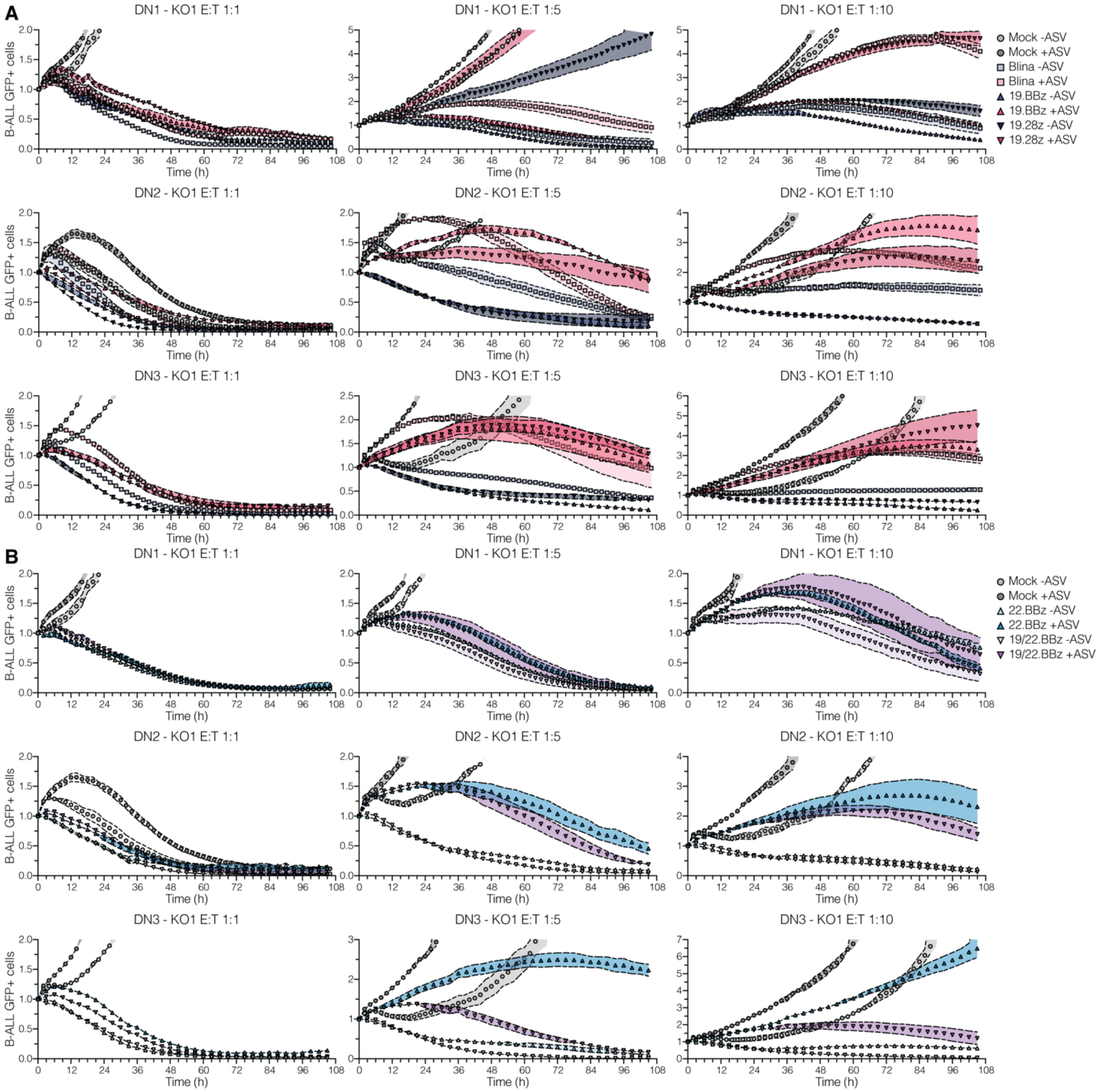
A
DN1 - KO1 E:T 1:1
DN1 - KO1 E:T 1:5
DN1 - KO1 E:T 1:10
DN2 - KO1 E:T 1:1
DN2 - KO1 E:T 1:5
DN2 - KO1 E:T 1:10
DN3 - KO1 E:T 1:1
DN3 - KO1 E:T 1:5
DN3 - KO1 E:T 1:10
B-ALL GFP+ cells
Time (h)
Mock -ASV
Mock +ASV
Blina -ASV
Blina +ASV
19.BBz -ASV
19.BBz +ASV
19.28z -ASV
19.28z +ASV
B
DN1 - KO1 E:T 1:1
DN1 - KO1 E:T 1:5
DN1 - KO1 E:T 1:10
DN2 - KO1 E:T 1:1
DN2 - KO1 E:T 1:5
DN2 - KO1 E:T 1:10
DN3 - KO1 E:T 1:1
DN3 - KO1 E:T 1:5
DN3 - KO1 E:T 1:10
Mock -ASV
Mock +ASV
22.BBz -ASV
22.BBz +ASV
19/22.BBz -ASV
19/22.BBz +ASV

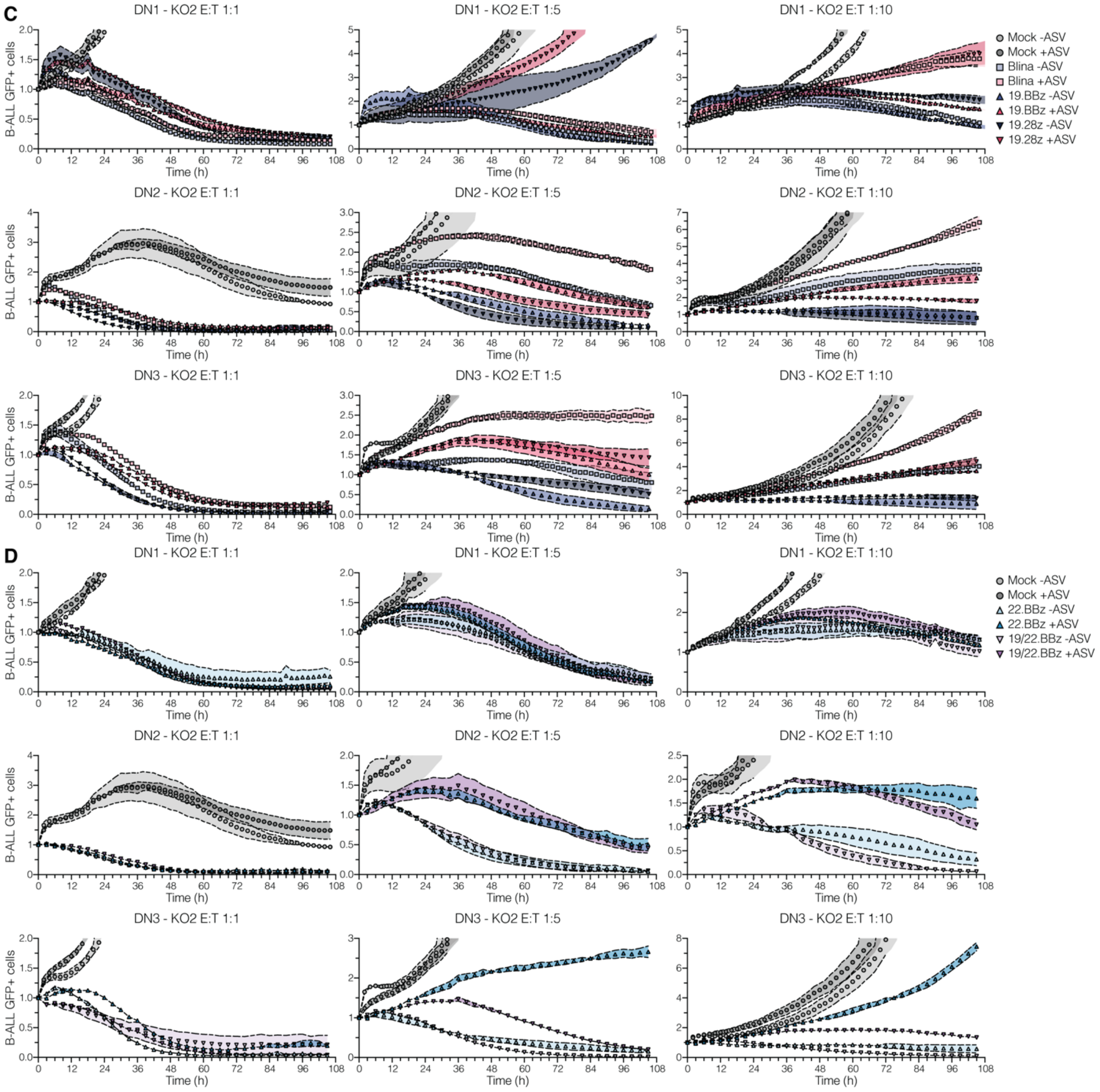

Supplementary Figure 9: Low IKAROS levels confer resistance against CD19- and CD22-targeted therapies.

(A - D) IKAROS-degron models (KO1, A - B; and KO2, C - D) were treated with DMSO (-ASU) or 10 µM asunaprevir (+ASU) for 7 days, before being co-cultured with mock, blinatumomab-treated, 19.BBz, and 19.28z (A or C) CAR T cells; or mock, 22.BBz, and dual 19/22.BBz (B or D) CAR T cells at 1:1, 1:5, and 1:10 E:T ratio. Experiment was conducted in triplicate, with different T cell donors for each replicate. B-ALL cell viability was measured at every 2 - 3 h interval via IncuCyte. GFP median values were normalized to 0 h condition. RFI = relative fluorescence intensity. Curves in (A - D) show mean ± SEM. Dots represent the mean value from three technical replicates.

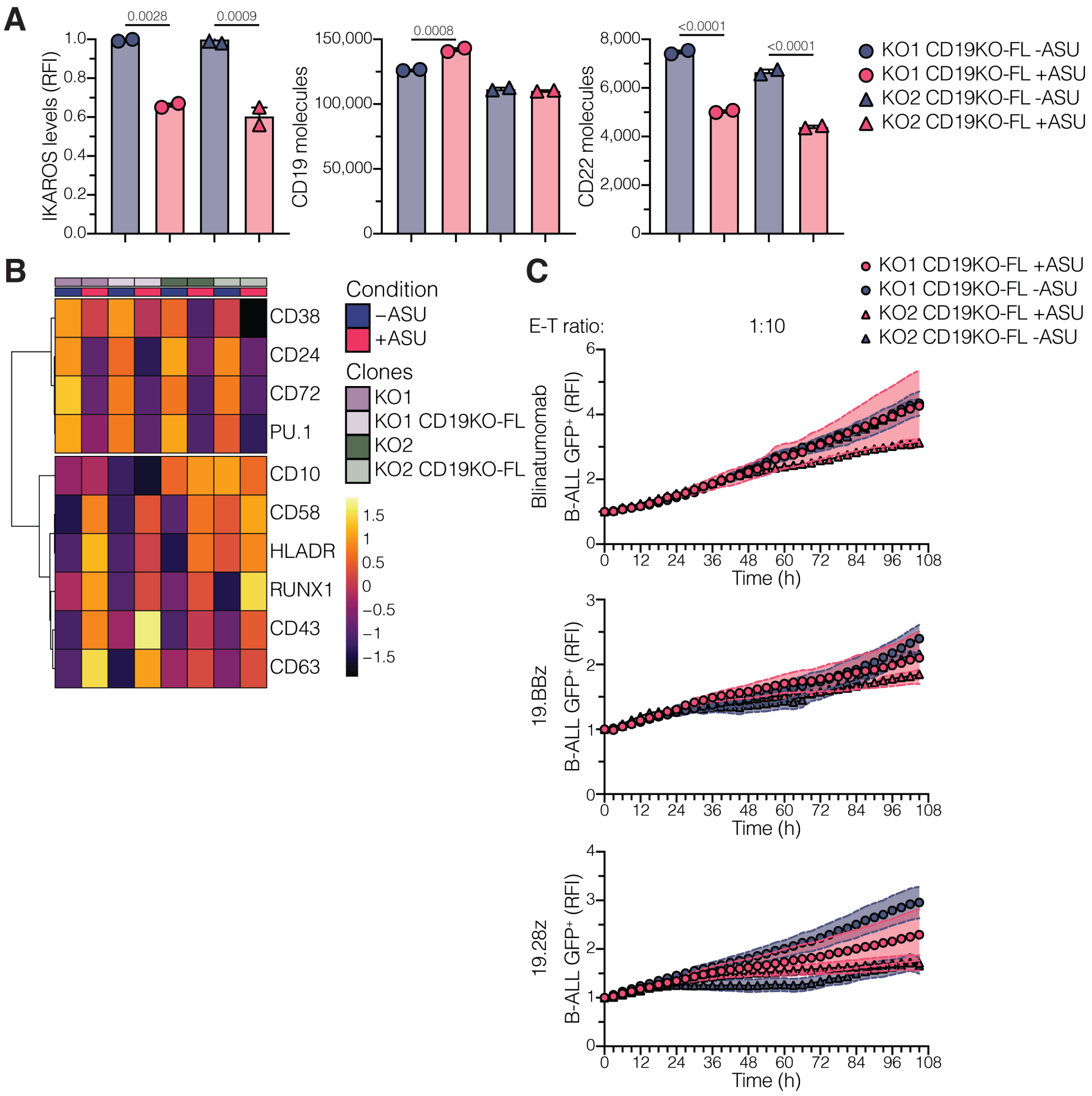

A
IKAROS levels (RFI)
0.0028
0.0009
CD19 molecules
0.0008
CD22 molecules
<0.0001
<0.0001
KO1 CD19KO-FL -ASU
KO1 CD19KO-FL +ASU
KO2 CD19KO-FL -ASU
KO2 CD19KO-FL +ASU
B
CD38
CD24
CD72
PU.1
CD10
CD58
HLADR
RUNX1
CD43
CD63
Condition
−ASU
+ASU
Clones
KO1
KO1 CD19KO-FL
KO2
KO2 CD19KO-FL
C
E-T ratio:
1:10
Blinatumomab
19.BBz
19.28z
B-ALL GFP+ (RFI)
Time (h)

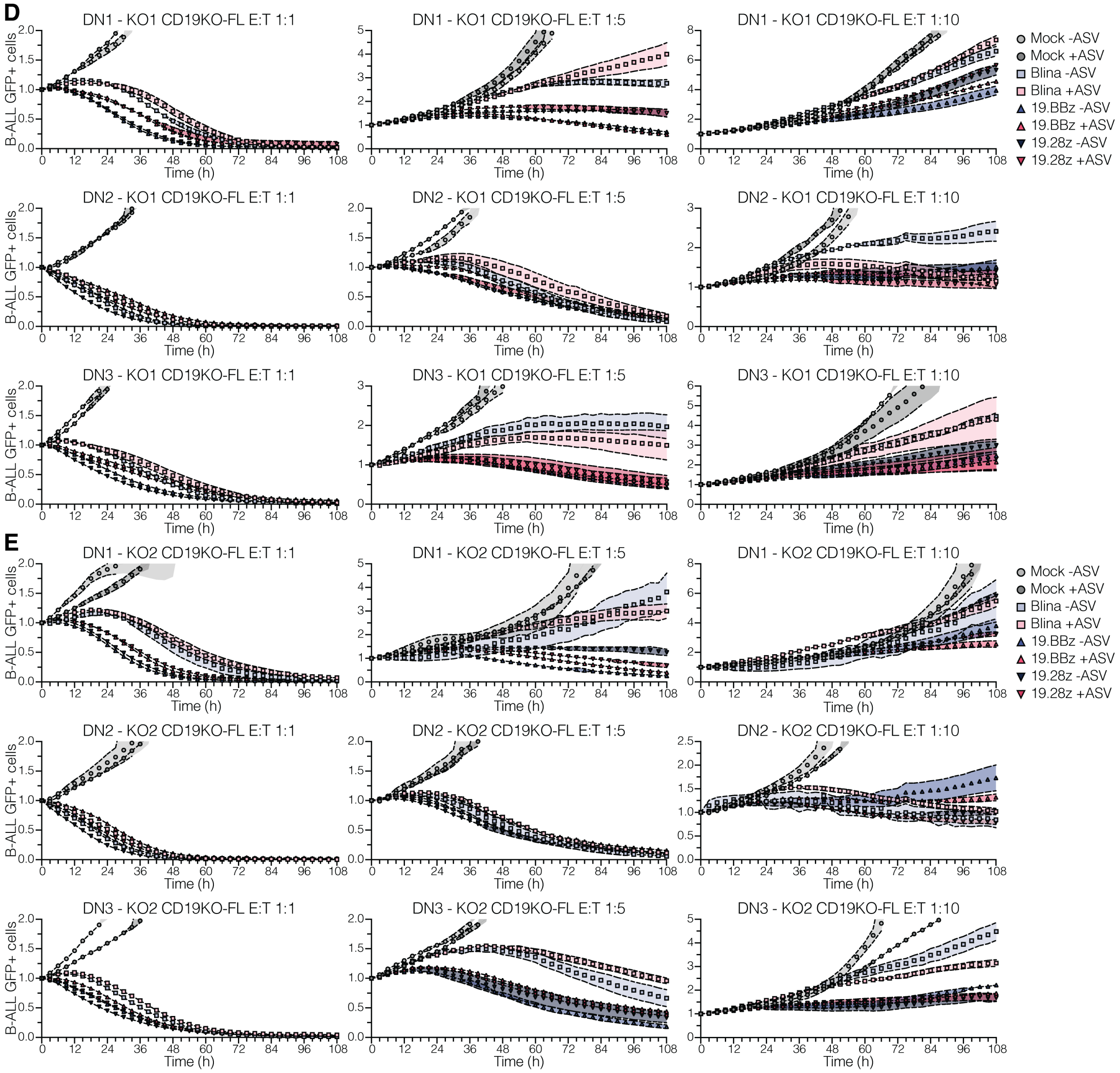

Supplementary Figure 10: IKAROS depends on CD19 modulation to confer resistance to CD19-targeted therapies.

(A) Relative IKAROS median levels (left), CD19 (middle), and CD22 (right) molecule numbers in IKAROS-degron CD19KO-FL model treated with DMSO (-ASU) or 10 µM asunaprevir (+ASU) for 7 days. Values were measured by flow cytometry. IKAROS median values were normalized to -ASU condition. RFI = relative fluorescence intensity.

(B) Median expression of key proteins in IKAROS-degron and IKAROS-degron CD19KO-FL models treated with DMSO (-ASU) or 10 µM asunaprevir (+ASU) for 7 days.

(C) IKAROS-degron CD19KO-FL models treated with DMSO (-ASU) or 10 µM asunaprevir (+ASU) for 7 days, and co-cultured with blinatumomab-treated (up), 19.BBz (middle), and 19.28z (bottom) CAR T cells at 1:10 E:T ratio.

(D - E) IKAROS-degron CD19KO-FL models (KO1, D; and KO2, E) treated with DMSO (-ASU) or 10 µM asunaprevir (+ASU) for 7 days, and co-cultured with mock, blinatumomab-treated,

19.BBz, and 19.28z CAR T cells at 1:1, 1:5, and 1:10 E:T ratio. Experiment was conducted in triplicate, with different T cell donors for each replicate. B-ALL cell viability was measured at every 2 - 3 h interval via IncuCyte. GFP median values were normalized to 0 h condition. RFI = relative fluorescence intensity.

Bar plots in (A) show mean ± SEM. Curves in (C - E) show mean ± SEM. Dots represent the mean value from three technical replicates. Statistical tests used were one-way ANOVA followed by Tukey's multiple comparisons tests (A); and two-way ANOVA followed by Šidák's multiple comparisons test (C - E).

Supplementary Table 1: Clinical information for patients in the CART19 and CART22 cohort.

CART19 cohort						
**Clinical outcome**	**pre-CART19 samples (n)**	**paired post-CART19 samples (n)**	**Age at treatment (yr) Median (Min - Max)**	**Gender (F/M)**	**Previous CD19-targeted therapy (Y/N)**	**Cytogenetics pre-CART19**
Complete remission (CR)	6	NA	12.5 (9 - 17)	4/2	1/5	CR1: Others<br>CR2: Trisomy 21<br>CR3: iAMP21<br>CR4: Hyperdiploid<br>CR5: Ph+<br>CR6: iAMP21
CD19neg relapse (Negative)	11	8	9 (6 - 43)	8/11	4/11	Neg1: Others<br>Neg2: ETV6-RUNX1<br>Neg3: Unknown<br>Neg4: Others<br>Neg5: ETV6-RUNX1<br>Neg6: Trisomy 21<br>Neg7: Ph+<br>Neg8: Hyperdiploid<br>Neg9: iAMP21<br>Neg10: Others<br>Neg11: Unknown
CD19pos relapse (Positive)	4	2	12.5 (6 - 38)	2/4	0/4	Pos1: Unknown<br>Pos2: Hypodiploid<br>Pos3: ETV6-RUNX1<br>Pos4: Hyperdiploid
Non responder (Refractory)	4	4	12 (5 - 21)	2/4	0/4	Ref1: Others<br>Ref2: Ph-like<br>Ref3: IKZF1 deletion<br>Ref4: IKZF1 deletion

CART22 cohort						
**Clinical outcome**	**pre-CART22 samples (n)**	**paired post-CART22 samples (n)**	**Age at treatment (yr) Median (Min - Max)**	**Gender (F/M)**	**Previous CD19-targeted therapy (Y/N)**	**Previous CD22-targeted therapy (Y/N)**
CD22low relapse	11	11	14 (4 - 31)	3/8	11/0	7/4

Supplementary Table 2: List of antibodies used for CyTOF experiments.

Antigen	Staining[(1)]	Metal	Isotope	Clone	Vendor	Concentration (µg/ml)	Panel
CD45	S	Y	89	HI30	BioLegend	0.5	1 and 2
mouse CD45	S	In	113	30-F11	BioLegend	0.25	1
CD61	S	In	115	VI-PL2	BioLegend	1	1 and 2
CD235ab	S	In	115	HIR2	BioLegend	1	1 and 2
cleaved PARP	I	La	139	F21-852	BD Biosciences	0.5	1 and 2
CD138	S	Ce	140	DL-101	BioLegend	4	1
CD63	S	Ce	140	H5C6	BioLegend	1	2
SRC (pY418)	I	Pr	141	K98-37	BD Biosciences	1	1
PLCγ2 (pY759)	I	Pr	141	K86-689.37.73	BD Biosciences	1	2
CD19	S	Nd	142	HIB19	BioLegend	0.25	1 and 2
CD22	S	Nd	143	HIB22	BioLegend	1	1 and 2
4EBP1 (pT37/46)	I	Nd	144	236B4	Cell Signaling Technology	0.5	1
RUNX1	I	Nd	144	Polyclonal (4334)	Cell Signaling Technology	8	2
IKAROS	I	Nd	145	D10E5	Cell Signaling Technology	2	1 and 2
PAX5	I	Nd	146	1H9	BioLegend	1	1 and 2
CD20	S	Sm	147	2H7	BioLegend	1	1 and 2
CD3	S	Nd	148	UCHT1	BioLegend	0.25	1 and 2
NFkB (pS529)	I	Sm	149	K10-895.12.50	BD Biosciences	0.5	1 and 2
STAT5 (pY694)	I	Nd	150	47/Stat5(pY694)	BD Biosciences	0.5	1
CD33	S	Nd	150	WM53	BioLegend	2	2
IgMs	S	Eu	151	MHM-88	BioLegend	0.5	1 and 2
Ki67	I	Sm	152	B56	BD Biosciences	2	1 and 2
IgMi	I	Eu	153	Polyclonal (NBP175017)	Novus Biologicals	1	1 and 2
HLA-DR	S	Sm	154	L243	BioLegend	1	1 and 2
CD13	S	Gd	155	WM15	BioLegend	1	1
CD58	S	Gd	155	LFA-3	BioLegend	2	2
CD10	S	Gd	156	HI10a	BioLegend	0.5	1 and 2
PU.1	I	Gd	157	9G7	Cell Signaling Technology	1	1 and 2
CD179b	I	Gd	158	HSL11	BioLegend	2	1 and 2
AKT (pS473)	I	Tb	159	D9E	Cell Signaling Technology	1	1
mNeonGreen	I	Tb	159	E8E3V	Cell Signaling Technology	1	2
CD27	S	Gd	160	O323	BioLegend	1	1 and 2
CD81	S	Dy	161	5A6	BioLegend	0.4	1
cleaved Caspase3	I	Dy	161	C92-605	BD Biosciences	1	2
GCR	I	Dy	162	D8H2	Cell Signaling Technology	2	1 and 2
CD127	S	Dy	163	A019D5	BioLegend	4	1
STAT3 (pY705)	I	Dy	163	4/P-STAT3	BD Biosciences	2	2
TdT	I	Dy	164	E17-1519	BD Biosciences	2	1 and 2
TSLPR	S	Ho	165	1B4	BioLegend	0.5	1
CREB (pS133)	I	Ho	165	87G3	Cell Signaling Technology	2	2
SYK (pY352)	I	Er	166	17A/P-ZAP70	BD Biosciences	0.5	1 and 2
BID	I	Er	167	Polyclonal (PA5-11384)	Invitrogen	4	1
CD43	S	Er	167	CD43-10G7	BioLegend	1	2
CD38	S	Er	168	HIT2	BioLegend	1	1 and 2
P38 (pT180/pY182)	I	Tm	169	36/p38 (pT180/pY182)	BD Biosciences	0.5	1 and 2
FADD	I	Er	170	EPR4415	Abcam	4	1
CD72	S	Er	170	3F3	BioLegend	2	2
CD24	S	Yb	171	ML5	BioLegend	1	1 and 2
S6 (pS235/pS236)	I	Yb	172	N7-548	BD Biosciences	0.5	1 and 2
CD34	S	Yb	173	581	BioLegend	0.5	1 and 2
BTK (pY551)	I	Yb	174	24a/BTK (Y551)	BD Biosciences	0.5	1 and 2
c-MYC	I	Lu	175	9E10	BioLegend	4	1
PTBP1	I	Lu	175	EPR9048(B)	Abcam	0.25	2
CREB (pS133)	I	Yb	176	87G3	Cell Signaling Technology	0.5	1
CD81	S	Yb	176	5A6	BioLegend	0.5	2
CD68	S	Pt	194	Y1/82A	BioLegend	0.2	2
CD16	S	Bi	209	3G8	BioLegend	2	1
CD11b	S	Bi	209	ICRF44	BioLegend	2	1 and 2
CD33	S	Bi	209	WM53	BioLegend	2	1

Supplementary Table 3: List of antibodies used for Ab-seq experiments.

Antigen	Conjugate	Clone	Vendor	Dilution (µl/ M cells)
mouse CD45	Biotin	30F11	BioLegend	0.5
CD3	Biotin	UCHT1	BioLegend	1
CD16	Biotin	3G8	BioLegend	2
CD11b	Biotin	ICRF44	BioLegend	1
CD33	Biotin	WM53	BioLegend	0.5
CD61	Biotin	Y2/51	Miltenyi Biotec	1
CD235	Biotin	HIR2	BioLegend	0.016
CD19	Oligo AHS0161	HIB19	BD Biosciences	2
CD20	Oligo AHS0008	2H7	BD Biosciences	2
CD24	Oligo AHS0042	ML5	BD Biosciences	2
CD34	Oligo AHS0061	581	BD Biosciences	2
CD38	Oligo AHS0022	HIT2	BD Biosciences	2
CD45	Oligo AHS0040	HI30	BD Biosciences	2
CD127	Oligo AHS0028	HIL-7R-M21	BD Biosciences	2
IgM	Oligo AHS0198	G20-127	BD Biosciences	2
