# Supplementary material for: IKAROS levels are associated with antigen escape in CD19- and CD22-targeted therapies for B-cell malignancies
Source: Nat Commun. 2025 Apr 23;16:3800. doi: 10.1038/s41467-025-58868-2 (PMC12019336; doi:10.1038/s41467-025-58868-2)
Supplement: Supplementary file 2 — Description of Additional Supplementary Information [file 41467_2025_58868_MOESM2_ESM.docx]

**Description of Additional Supplementary Files**

**Supplementary Data** **1:** List of differentially expressed genes in healthy BM clusters. Two-sided Wilcoxon rank sum test followed by Bonferroni’s multiple comparisons.

**Supplementary Data** **2:** List of differentially expressed genes between pre-CART19 CR or CD19^neg^ relapse pro-B like B-ALL cells. Two-sided Wilcoxon rank sum test followed by Bonferroni’s multiple comparisons.

**Supplementary Data** **3:** List of differentially accessible peaks between IKAROS WT and KD B-ALL cells. DESeq’s Wald test followed by BH correction.

**Supplementary Data** **4:** List of differentially expressed genes between IKAROS WT and KD B-ALL cells. DESeq’s Wald test followed by BH correction.
