# Supplementary material for: IKAROS levels are associated with antigen escape in CD19- and CD22-targeted therapies for B-cell malignancies
Source: Nat Commun. 2025 Apr 23;16:3800. doi: 10.1038/s41467-025-58868-2 (PMC12019336; doi:10.1038/s41467-025-58868-2)
Supplement: Supplementary file 7 — Reporting Summary [file 41467_2025_58868_MOESM7_ESM.pdf]

# nature portfolio

Corresponding author(s): Kara L Davis, DO
Last updated by author(s): Aug 28, 2024

# Reporting Summary

Nature Portfolio wishes to improve the reproducibility of the work that we publish. This form provides structure for consistency and transparency in reporting. For further information on Nature Portfolio policies, see our Editorial Policies and the Editorial Policy Checklist.

## Statistics

For all statistical analyses, confirm that the following items are present in the figure legend, table legend, main text, or Methods section.

| n/a | Confirmed | |
|---|---|---|
| ☐ | ☒ | The exact sample size (*n*) for each experimental group/condition, given as a discrete number and unit of measurement |
| ☐ | ☒ | A statement on whether measurements were taken from distinct samples or whether the same sample was measured repeatedly |
| ☐ | ☒ | The statistical test(s) used AND whether they are one- or two-sided<br>*Only common tests should be described solely by name; describe more complex techniques in the Methods section.* |
| ☒ | ☐ | A description of all covariates tested |
| ☐ | ☒ | A description of any assumptions or corrections, such as tests of normality and adjustment for multiple comparisons |
| ☐ | ☒ | A full description of the statistical parameters including central tendency (e.g. means) or other basic estimates (e.g. regression coefficient) AND variation (e.g. standard deviation) or associated estimates of uncertainty (e.g. confidence intervals) |
| ☐ | ☒ | For null hypothesis testing, the test statistic (e.g. *F*, *t*, *r*) with confidence intervals, effect sizes, degrees of freedom and *P* value noted<br>*Give P values as exact values whenever suitable.* |
| ☒ | ☐ | For Bayesian analysis, information on the choice of priors and Markov chain Monte Carlo settings |
| ☒ | ☐ | For hierarchical and complex designs, identification of the appropriate level for tests and full reporting of outcomes |
| ☒ | ☐ | Estimates of effect sizes (e.g. Cohen's *d*, Pearson's *r*), indicating how they were calculated |

*Our web collection on statistics for biologists contains articles on many of the points above.*

## Software and code

Policy information about availability of computer code

| | |
|---|---|
| Data collection | CytExpert (Beckman Coulter): Flow cytometry data acquisition.<br>Helios Instrument Control Software version 7.0.8493 (Fluidigm): Mass cytometry data acquisition.<br>NovaSeq Control Software (Illumina): bulk RNA-seq, ATAC-seq and single-cell CiTE-seq data acquisition.<br>IncuCyte S3 ver 2019B Rev2 Software (Sartorius): Cytotoxicity data acquisition<br>BD FACSDiva™ Software v. 8.0.1 (BD Biosciences): Cell sorting data acquisition |
| Data analysis | Community cytobank (https://community.cytobank.org/cytobank/): Flow cytometry and Mass cytometry data analysis.<br>Rhapsody analysis pipeline (BD Biosciences): Single-cell CiTE-seq data analysis.<br>R version 4.0.2: Mass cytometry, single-cell CiTE-seq, bulk RNA-seq and ATAC-seq data analysis. Generation of graphs and statistical analysis.<br>Developmental classifier R code (https://github.com/kara-davis-lab/DEVclassifier): To developmental classify mass cytometry data.<br>Seurat R package (version 4.2.0): single-cell CiTE-seq data analysis.<br>Umap R package (version 0.2.9.0): To generate umap plots from mass cytometry and single-cell CiTE-seq data.<br>ComplexHeatmap R package (version 2.6.2): To generate heatmap plots from mass cytometry and single-cell CiTE-seq data.<br>SALMON (version 1.2.0): Alignment of bulk RNA-seq data.<br>STAR (version 2.7.8a ): Alignment of bulk RNA-seq data.<br>Bowtie2 (version 2.2.6): Alignment of ATAC-seq data.<br>DESeq2 R package (version 1.30.1): Differential analysis of bulk RNA-seq and ATAC-seq data.<br>GSEA (version 4.2.2): Gene set enrichment analysis for bulk RNA-seq data.<br>EnrichR (https://maayanlab.cloud/Enrichr/): Pathway and cell type enrichment analysis for bulk RNA-seq, ATAC-seq and single-cell CiTE-seq |

data.
rMATS (version 4.1.1): Multivariate analysis of transcript splicing for bulk RNA-seq data.
IGV software (version 2.8.10): Visualizacion ATAC-seq peaks.
GraphPad Prism software (version 9.3.1): Generation of graphs and statistical analysis.
SnapGene ver 7.2.1 (Dotmatics): DNA vector design and molecular cloning.

For manuscripts utilizing custom algorithms or software that are central to the research but not yet described in published literature, software must be made available to editors and reviewers. We strongly encourage code deposition in a community repository (e.g. GitHub). See the Nature Portfolio guidelines for submitting code & software for further information.

## Data

Policy information about availability of data

All manuscripts must include a data availability statement. This statement should provide the following information, where applicable:

- Accession codes, unique identifiers, or web links for publicly available datasets
- A description of any restrictions on data availability
- For clinical datasets or third party data, please ensure that the statement adheres to our policy

Mass cytometry data from clinically annotated patient samples from the CART19 cohort, CART22 cohort, and IKAROS-degron, IK6-degron, IKAROS-degron CD19KO-FL cell models are available on Community Cytobank under accession numbers 121064 [https://community.cytobank.org/cytobank/experiments/121064], 121078 [https://community.cytobank.org/cytobank/experiments/121078], and 121091 [https://community.cytobank.org/cytobank/experiments/121091], respectively.

Bulk RNA-seq and ATAC-seq data from isogenic IKAROS WT or KD B-ALL cell lines have been deposited in NCBI Gene Expression Omnibus (GEO) and are accessible through GEO SuperSeries accession number GSE225755 [https://www.ncbi.nlm.nih.gov/geo/query/acc.cgi?&acc=GSE225755].

Final expression matrices containing recursive substation error correction (RSEC) adjusted molecule counts per cell for single-cell CITE-seq data from PDXs are available in Mendeley data (doi:10.17632/x6kkjp7xyv.1) [https://data.mendeley.com/preview/x6kkjp7xyv?a=34e55122-a9ef-406a-bf5f-e27980ee0ca1]

Therapeutically Applicable Research to Generate Effective Treatments (TARGET) dataset, phs000218, used for this study, is available at https://portal.gdc.cancer.gov/projects. BAM files with RNA seq reads aligned to the CD19 gene from Orlando, E. et al. can be found in the NCBI Sequence Read Archive (SRA) at SRP141691. Raw RNA-seq fastq files from Plaks, V. et al. can be found under accession number PRJNA727804. Raw RNA-seq fastq files from Sotillo, E. et al., Zhao, Y. et al., and Sworder, B. et al. were obtained after request to the corresponding authors. Raw direct RNA long-reads from one B-ALL PDX is available in the SRA, at accession number SRR14326969.

## Human research participants

Policy information about studies involving human research participants and Sex and Gender in Research.

| | |
|---|---|
| Reporting on sex and gender | This information has been reported in Supplementary Table 1. All the analyses performed were not gender biased. |
| Population characteristics | In Supplementary Table 1, for each patient treated with CD19-directed CAR T cells, genetic background, previous CD19-directed therapies, age at treatment, cytokine release syndrome grade, time of CAR T cells persistance, time to relapse and clinical response to CD19-directed CAR T cells have been reported, when available.<br>In Supplementary Table 8, for each patient treated with CD22-directed CAR T cells, disease burden at time of sample collection, genetic background, previous CD19-, CD20-, and CD22-targeted therapies, age at treatment, previous number of relapses, cytokine release syndrome grade, time to relapse and clinical response to CD22-directed CAR T cells have been reported, when available |
| Recruitment | Samples were obtained from patients enrolled in NCT01626495 (n = 18), NCT02228096 (n = 4), NCT02906371 (n = 1), and NCT02315612 (n = 11) clinical trials. Samples from two patients that were treated compassionately were also collected. The samples were collected as part of IRB-approved clinical research studies. |
| Ethics oversight | The Institutional Review Board (IRB) of Stanford University approved the use of these primary samples for this work. |

Note that full information on the approval of the study protocol must also be provided in the manuscript.

# Field-specific reporting

Please select the one below that is the best fit for your research. If you are not sure, read the appropriate sections before making your selection.

[x] Life sciences  [ ] Behavioural & social sciences  [ ] Ecological, evolutionary & environmental sciences

For a reference copy of the document with all sections, see nature.com/documents/nr-reporting-summary-flat.pdf

# Life sciences study design

All studies must disclose on these points even when the disclosure is negative.

| | |
|---|---|
| Sample size | Sample size of primary samples was determined based on availability of samples with sufficient material and annotated clinical data. |
| Data exclusions | No data were excluded from the analysis. |
| Replication | Patient samples analyzed by mass cytometry or single-cell CITE-seq were not performed in replicated. Different healthy BM samples were used for the following mass cytometry experiments, with replicates of the same healthy BM within the same experiment: PDX samples from patients treated with CD19-directed CAR T cells, isogenic CD19 WT or KO B-ALL cell lines, primary samples from patients treated with CD22-directed CAR T cells, IKAROS-degron time-course experiment and comparison with IKAROS-degron CD19KO-FL models, IKAROS-degron vs. IK6-degron models comparison. Replicates from the same healthy BM sample were used for single-cell CITE-seq. Isogenic IKAROS WT and KD ATAC-seq and RNA-seq experiments were performed with 3 different cell lines, each one of them in duplicate. For flow cytometry and mass cytometry experiments with cell lines, the respective number of cell lines and replicates are described in the corresponding figure legends. For analysis of isogenic CD19 WT and KO B-ALL cells, 4 different B-ALL cell lines were used without technical replicate. For analysis of IKAROS KD and/or lenalidomide treatment, 7 B-ALL, 3 LBCL, and 3 CLL cell lines were used, and each one of them was at least in duplicate. For IKAROS-degron, IK6-degron, and IKAROS-degron CD19KO-FL models, 7, 3, and 2 clones were characterized, respectively. IncuCyte killing assays were performed in triplicate, with 3 different T cell donors, and with 2 different IKAROS-degron or IKAROS-degron CD19KO-FL clones. |
| Randomization | Randomization were not required in any experiment from this study |
| Blinding | Primary patient samples were run in different plates and the label assignment was required to investigate batch effect. Therefore, blinding was not possible for such analysis. Flow cytometry were performed by the same investigator so blinding was not possible. |

# Reporting for specific materials, systems and methods

We require information from authors about some types of materials, experimental systems and methods used in many studies. Here, indicate whether each material, system or method listed is relevant to your study. If you are not sure if a list item applies to your research, read the appropriate section before selecting a response.

## Materials & experimental systems

| n/a | Involved in the study | |
|---|---|---|
| ☐ | ☒ | Antibodies |
| ☐ | ☒ | Eukaryotic cell lines |
| ☒ | ☐ | Palaeontology and archaeology |
| ☒ | ☐ | Animals and other organisms |
| ☐ | ☒ | Clinical data |
| ☒ | ☐ | Dual use research of concern |

## Methods

| n/a | Involved in the study | |
|---|---|---|
| ☒ | ☐ | ChIP-seq |
| ☐ | ☒ | Flow cytometry |
| ☒ | ☐ | MRI-based neuroimaging |

## Antibodies

| | |
|---|---|
| Antibodies used | All the antibodies used for mass cytometry and CITE-seq experiments with informations regarding clone, vendor, conjugated and concentrations used are described in Supplementary Table 2 and 3, respectively.<br>For flow cytometry antibodies:<br>Antigen, clone, manufacturer, fluorophore, concentration used (per 100 µl reaction), cat number<br>- CD19, HIB19, Biolegend, APC, 1 uL, 302212.<br>- CD19, HIB19, Biolegend, Pacific Blue, 1 uL, 302232.<br>- CD22, HIB22, Biolegend, APC, 1 uL, 302510.<br>- IKAROS, 16B5C71, BioLegend, Alexa Flour 647, 1 µl, 368404 |
| Validation | All antibodies were validated in human cells (cell lines or primary cells) known to be positive or negative controls for a given antibody target. Each antibody was titrated in concentration range from 0.5 to 8 ug/mL and the lowest concentration that discriminate positive from negative without spill over in another channels was chosen. |

## Eukaryotic cell lines

Policy information about cell lines and Sex and Gender in Research

| | |
|---|---|
| Cell line source(s) | ATCC: NALM6 (CRL-3273), REH (CRL-8286), RS4;11 (CRL-1873), SUP-B15 (CRL-1929)<br>DSMZ: 697 (ACC 42), CI (ACC 770), JVM-2 (ACC 12), MHH-CALL4 (ACC 337), NALM16 (ACC 680), NALM20 (ACC 681), WA-OSEL (ACC 767)<br>Amengual lab: OCI-Ly1, OCI-Ly-7, and SUDHL6 |

| | |
|---|---|
| Authentication | Cells lines were authenticated by the suppliers or by STR profiling in January 2019 and early passages were cryopreserved. For the experiments they were used within 5 to 10 passages. |
| Mycoplasma contamination | Cell lines were routinely tested for Mycoplasma contamination before in vitro experiments using MycoAlert Mycoplasma Detection kit from Lonza and were confirmed to be negative. |
| Commonly misidentified lines (See ICLAC register) | No commonly misidentified cell lines were used. |

# Clinical data

Policy information about clinical studies

All manuscripts should comply with the ICMJEguidelines for publication of clinical research and a completedCONSORT checklist must be included with all submissions.

| | |
|---|---|
| Clinical trial registration | NCT01626495, NCT02228096, NCT02906371, NCT02315612 |
| Study protocol | The full protocols are accessible on Clinicaltrials.gov. |
| Data collection | For CD19-directed CAR T patient, primary samples with available material were collected at the time of apheresis or after CART19 relapse. These samples were engrafted in immune deficient mice and PDXs samples from these patients were used for this study. For CD22-directed CAR T patient, primary samples with available material were collected at the time of apheresis or after CD22low relapse. These samples were viable frozen and used for this study. |
| Outcomes | We did not report any outcome status for this work. |

# Flow Cytometry

## Plots

Confirm that:

- [x] The axis labels state the marker and fluorochrome used (e.g. CD4-FITC).
- [x] The axis scales are clearly visible. Include numbers along axes only for bottom left plot of group (a 'group' is an analysis of identical markers).
- [x] All plots are contour plots with outliers or pseudocolor plots.
- [x] A numerical value for number of cells or percentage (with statistics) is provided.

## Methodology

| | |
|---|---|
| Sample preparation | To assess CD19 and CD22 surface expression, 0.5 – 1 x 105 cells were washed with CSM and incubated with 100 µl antibody mix (1 µl APC anti-human CD19 (clone: HIB19, BioLegend); or 1 µl APC anti-human CD22 (clone: HIB22, BioLegend) with or without 1 µl Pacific Blue anti-human CD19 (clone: HIB19, BioLegend) in 100 µl CSM) at RT for 10 min. Cells were washed twice and resuspended in 200 µl CSM for flow analysis in CytoFLEX cytometer (Beckman Coulter).<br>To assess IKAROS intracellular levels, 0.5 – 1 x 105 cells were washed with CSM and fixed with 1.6% PFA (Electron Microscopy Sciences) in CSM for 10 min at room temperature (RT). Cells were washed twice with CSM and incubated with 1 µl Pacific Blue anti-human CD19 antibody (clone: HIB19, BioLegend) in 100 µl CSM at RT for 10 min. Cells were washed twice and permeabilized with 100 µl Methanol at 4ºC for 10 min. After three washes, cells were incubated with 1 µl Alexa Flour 647 anti-human IKAROS antibody (clone: 16B5C71, BioLegend) in 100 µl CSM at RT and 300 rpm for 30 min. Cells were washed twice and resuspended in 200 µl CSM for flow analysis in CytoFLEX cytometer (Beckman Coulter).<br>Quantification of CD19 and CD22 surface molecules on cancer lines was performed using the BD Quantibrite™ APC Fluorescence Quantitation Kit (BD Biosciences). |
| Instrument | Flow cytometry data was collected in CytoFLEX cytometer (Beckman Coulter). |
| Software | Data was collected using CytExpert software, and was analyzed using Community cytobank (https://community.cytobank.org/cytobank/). |
| Cell population abundance | N/A |
| Gating strategy | Flow cytometry data was analyzed using Community Cytobank software (https://community.cytobank.org/). Briefly, forward versus side scatter was used to exclude debris, while forward scatter area versus width was used for doublet exclusion. When pertinent, tumor cells were gated based on GFP (IKAROS-degron, IKAROS-degron CD19KO-FL, IK6-degron, and CD19 isoforms overexpressing cells) or RFP (IKAROS WT and KD cells) expression. Finally, CD19, CD22, and IKAROS median fluorescent intensities (MFI) were calculated in the gated population. |

- [x] Tick this box to confirm that a figure exemplifying the gating strategy is provided in the Supplementary Information.
